# Individual Differences in the Neural and Cognitive Mechanisms of Single Word Reading

**DOI:** 10.3389/fnhum.2018.00271

**Published:** 2018-07-05

**Authors:** Simon Fischer-Baum, Jeong Hwan Kook, Yoseph Lee, Aurora Ramos-Nuñez, Marina Vannucci

**Affiliations:** ^1^Department of Psychology, Rice University, Houston, TX, United States; ^2^Department of Statistics, Rice University, Houston, TX, United States; ^3^Department of Social Sciences, Coastal College of Georgia, Brunswick, GA, United States

**Keywords:** fMRI, visual word recognition, reading aloud, sublexical processing, individual differences

## Abstract

Written language is a human invention that our brains did not evolve for. Yet, most research has focused on finding a single theory of reading, identifying the common set of cognitive and neural processes shared across individuals, neglecting individual differences. In contrast, we investigated variation in single word reading. Using a novel statistical method for analyzing heterogeneity in multi-subject task-based functional magnetic resonance imaging (fMRI), we clustered readers based on their brain’s response to written stimuli. Separate behavioral testing and neuroimaging analysis shows that these clusters differed in the role of the sublexical pathway in processing written language, but not in reading skill. Taken together, these results suggest that individuals vary in the cognitive and neural mechanisms involved in word reading. In general, neurocognitive theories need to account not only for what tends to be true of the population, but also the types of variation that exist, even within a neurotypical population.

## Introduction

Cognitive scientists typically assume individuals rely on a common set of cognitive operations with essentially the same neural organization (de Schotten and Shallice, 2017). At the same time, there is clear variability across individuals, in terms of skills, experiences and abilities. Understanding both general patterns of cognitive and brain functioning and the ways that individual subjects depart from those general patterns is critical for understanding how the human mind works.

The current project focuses on variability in the cognitive and neural processes involved in reading. Reading is a particularly fruitful topic for investigating neuro-variability. Written language is a relatively recent human invention, and therefore reading is not something that our brains evolved to do. Unlike spoken language, literacy skills are learned through explicit instruction, with different learning techniques that have been proposed (Rayner et al., 2002). Yet, individuals from a wide variety of linguistic and educational backgrounds have been argued to rely on essentially the same reading system, both in terms of cognitive architectures (Perfetti, 2011) and neural substrates (Rueckl et al., 2015). This extends even to readers of vastly different writing systems, with similar brain regions involved in processing logographic languages, transparent and opaque alphabetic languages and even Braille which is read by touch rather than by sight (Reich et al., 2011; Rueckl et al., 2015). Despite the fact that reading is a taught skill that our brains did not evolve for, it appears that most people learn to do it approximately the same way.

At the same time, it is clear that readers vary, at least to some degree, in how they process written language. Even among highly skilled, literate adult readers without any evidence of reading difficulties like dyslexia, there are differences in reading skill (Andrews, 2012). These differences in reading skills have been mapped onto variability in the efficacy of different cognitive processes, like linking from letters to sound (Gough and Tunmer, 1986), recognizing familiar words (Perfetti, 2007; Andrews, 2012), comprehending spoken sentences (Gough and Tunmer, 1986) and knowledge about the phonological structure of words (Johnston and Kirby, 2006). Variability in reading skill has been linked to neural differences, for example with differences in sight word and phonemic decoding efficiency relating to different degrees of BOLD response to written words in temporal, occipital and parietal regions during functional MRI (Welcome and Joanisse, 2012), or differences in the semantic influences on reading relating to differences in the integrity of white matter pathways connecting regions within the left temporal lobe and between the superior temporal gyrus and angular gyrus (AG; Graves et al., 2014; see also Seghier et al., 2008, 2011; Kherif et al., 2009; Jobard et al., 2011; Welcome and Joanisse, 2014).

How does this variability relate to our understanding of how we read words? Most theories of word reading assume that there are multiple pathways for reading words aloud, with the lexical/semantic pathway focusing on processing the meaning and pronunciation associated with familiar words and a sublexical/phonological pathway with a sounding out procedure that maps directly from letters to sounds irrespective of whether or not the stimulus is a known word (Plaut et al., 1996; Coltheart et al., 2001). While both pathways are capable of generating correct word pronunciations, at least for familiar words with regular spelling to sound correspondences, these theories also assume that when readers are confronted with a word, both pathways are activated simultaneously and in parallel. However, just because both pathways are used to read does not mean that they carry equal weight. A variety of studies have demonstrated that the relative weight of the lexical and sublexical pathways can be manipulated strategically based on the makeup of words in the experiment (Monsell et al., 1992; Rastle and Coltheart, 1999; Zevin and Balota, 2000; see Lupker et al., 1997) or based on task demands (e.g., Bitan et al., 2005). Some researchers have proposed that the weight of these routes also varies as a function of individuals, with some participants depending more on lexical processes when they read and others depending more on sublexical processes (Baron and Strawson, 1976; Woollams et al., 2016), though this position remains a matter of debate (Brown et al., 1994; Yap et al., 2012). We explore that possibility here.

Our approach involves a combination of functional magnetic resonance imaging (fMRI), behavioral experiments designed to tap into phonological processing of written words, and standardized tests of reading skill. We take a novel, data-driven approach to analyzing multi-subject, task-based fMRI data (Zhang et al., 2014, 2016) that can cluster subjects into subgroups characterized by similar patterns of brain responses across the whole brain to written words. We then ask whether these subgroups differ in the engagement of sublexical and/or lexical processes on the basis of their performance on the behavioral tasks and on analyses of an orthogonal set of neuroimaging data. Previous studies have demonstrated that clustering techniques can be used to identify subgroups of readers on the basis of the neuronal activation for reading aloud (Kherif et al., 2009). However, these previous experiments collected only limited behavioral data, making it is difficult to interpret how these subgroups differ cognitively.

The current study involves a much richer set of behavioral and neuroimaging measures designed to evaluate lexical and sublexical processing. Behavioral tasks include classic effects in both lexical decision and reading aloud. The *pseudohomophone lexical decision* paradigm can be used to test phonological processing in the context of a lexical decision task. In this task, pseudowords are evenly divided between pseudohomophones, or pseudowords that are pronounced like real words (e.g., BRANE) and non-pseudohomophones, or matched pseudowords whose pronunciations do not correspond to familiar words (e.g., BRAME; Rubenstein et al., 1971; Besner and Davelaar, 1983). Participants are slower and make more errors when they have to reject pseudohomophones than non-pseudohomophones, which has been used to argue for an influence of sublexical processing on a task that could be carried out exclusively with lexical/semantic processing. In reading aloud experiments, researchers analyze the speed with which participants read different types of orthographic stimuli. The *lexicality* effect, or the difference in the speed with which pseudowords and familiar words are read, has been used to isolate sublexical processing ability, with smaller lexicality effects indicating stronger sublexical processing. The *regularity* effect, or the difference in the speed with which regular words (e.g., CLAM), that can be correctly sounded out by the sublexical route, and irregular words (e.g., YACHT), with idiosyncratic pronunciations that can only be correctly processed by the lexical route, are read has been used to isolate lexical processing ability, with smaller differences between these two word types indicating a greater reliance lexical/semantic processing. The size of the pseudohomophone effect, lexicality effect and regularity effect all vary, to some extent, by participant strategies (e.g., Coltheart and Rastle, 1994; Wagenmakers et al., 2008), but individual difference approaches have found clear relationships in performance on the two tasks, as well as clear links to individual variation in reading skill (Katz et al., 2012).

Neuroimaging has also been used to evaluate lexical and sublexical processing. A neurobiological distinction has been made between dorsal and ventral reading routes. Dorsal reading areas, including the parietal lobe and superior temporal gyrus have been linked to sublexical/phonological processing of written words, while ventral reading areas including ventral occipitotemporal and middle temporal gyrus regions have been linked to lexical/semantic processing (Schlaggar and McCandliss, 2007). In a recent meta-analysis, Taylor et al. (2013) argue that lexical and sublexical processes can be partially identified by comparing the BOLD response to word and pseudoword stimuli, with greater responses to pseudowords indicating sublexical engagement and greater response to words indicating lexical engagement. Using this approach, they identified dorsal regions, specifically in the inferior parietal lobule (IPL) and inferior frontal gyrus (IFG), as critical nodes in the sublexical reading pathway, and ventral regions, specifically the anterior fusiform and middle temporal gyrus, as critical nodes in the lexical reading pathway. The AG was also highlighted as part of the lexical pathway.

An alternative approach to evaluating the activation of semantic and phonological representations in different brain regions during reading tasks is the use of representational similarity analysis (RSA) fMRI (Fischer-Baum et al., 2017; Zhao et al., 2017). This approach can identify the type of information about written words represented in different cortical regions on the basis of the similarity of the fine-grained patterns of activity to different words. Consider the word DOUGH. DOUGH is related to TOUGH visually, SEW phonologically, and BREAD semantically. According to RSA logic, brain regions that have similar patterns of activity for DOUGH and SEW process phonology, rather than semantics or visual information while regions that have similar patterns of activity for DOUGH and BREAD process semantics.

Our study focuses on a sample of college students enrolled at a highly selective, 4-year university who have no previous or current diagnosis of dyslexia, dysgraphia or other learning difference. This sample is not representative of variation in reading ability across the entire population, as our participants are likely extremely skilled readers. To assess this, participants underwent a series of standardized measures of reading skill; the Nelson-Denny Reading Test (Form G; Brown et al., 1993), which was used to examine vocabulary knowledge, reading comprehension skill and reading rate and the Test of Word Reading Efficiency (TOWRE-2; Torgesen et al., 2012), which was used to examine decoding and sight-word reading ability. We focus on this population because neurotypical, English-speaking, college students are the most commonly studied population in the development of models of reading and are therefore the population that is typically assumed to be homogenous with respect to the processes involved in reading. It is of interest, therefore, to investigate whether there is variation in reading processes even within this population.

The study was divided into two sessions. In one session, participants read a combination of words and pseudowords aloud while undergoing fMRI scanning. In another session, participants were given a battery of behavioral tasks; standardized measures of reading skill (reading comprehension, reading rate, vocabulary knowledge, decoding ability), a pseudohomophone lexical decision study and a reading aloud experiment with pseudowords, regular words and irregular words. In order to avoid circular fMRI analyses (Kriegeskorte et al., 2009; Vul and Pashler, 2012), only data from the first run of the fMRI session was used to cluster participants into subgroups of readers on the basis of how their brains respond to written words. Data from the remaining five runs of the fMRI session were then used to compare how the brains of these two clusters of participants respond to different types of orthographic stimuli, both a whole brain univariate analysis compared groups in their response to words vs. pseudowords and a multivariate analysis using RSA to compare groups on how key regions of the reading network process semantic and phonological information about familiar words. Finally, these subgroups were compared in their performance on behavioral tasks, specifically the lexical decision task and the reading aloud task to determine if they differed with respect to size of their lexicality, regularity and pseudohomophone effect. Together, the neuroimaging and behavioral data suggest that the clustering algorithm identified two groups of reader that differ in how effortful the use of the sublexical/phonological route was in reading, despite no apparent differences in reading skill.

## Materials and Methods

### Participants

Thirty Rice University students (23 females; *M* = 19.73 years old; range 18–28 years) participated in the study. All were 18 years of age or older, Native English speaker, with no history of dyslexia, dysgraphia or neurological disorders and no contraindications to MRI. The participants were compensated $25 for each of the neuroimaging and the behavioral testing sessions. This study was carried out in accordance with the recommendations of the Rice University Institutional Review Board with written informed consent from all subjects and also in accordance with the Declaration of Helsinki.

### Behavioral Testing

Standardized measures of vocabulary knowledge, reading comprehension, reading rate and decoding ability were collected using the Nelson-Denny Reading Test and the TOWRE-2. The Nelson-Denny Reading Test is a standardized reading comprehension test for college level students and includes 80 multiple choice vocabulary questions and 38 reading comprehension questions. We also obtained a measure of reading rate (number of words read in the first minute of the reading comprehension section). For decoding ability, participants were administered two subtests of the TOWRE-2; Sight Word Efficiency (SWE), which measures the number of words that an individual can accurately identify within 45 s (108 maximum) and Phonemic Decoding Efficiency, which measures the number of pronounceable nonwords that an individual can accurately decode within 45 s (66 maximum). Participant’s performance on both the Nelson-Denny and the TOWRE-2 were compared to normative data.

Each participant was also administered a pseudohomophone lexical decision experiment. “Materials and Methods” section were taken directly from Besner and Davelaar (1983). The task had a total of 117 items that were categorized into three groups presented in random order: words (*n* = 39), nonwords that were pseudohomophones (*n* = 39), and nonwords that are non-pseudohomophones (*n* = 39), with a short practice session prior to the experimental trials. Nonword stimuli were matched on length in both number of letters and number of syllables and for number of orthographic neighbors. We used a smaller set of word stimuli than is typical in these experiments to save some time in the extensive behavioral testing session, meaning that there were twice as many nonwords than words. This modification may have reduced the size of the pseudohomophone effect, but as we show below, the effect is still present with this design. The experiment was presented on DmDx (Forster and Forster, 2003). A trial consisted of a fixation cross for 1500 ms followed by the experimental stimuli presented in uppercase, size 36 Times New Roman font. The word disappeared following 1500 ms or once a response was produced. The participants pressed the Left Shift key to indicate that the stimulus was not a word and the Right Shift key to indicate that the stimulus was a word and were instructed to respond as quickly and accurately as they could.

Finally, each participant was also administered a regular word, exception word and nonword reading experiment, also presented in DmDx. Materials were drawn from Baron and Strawson (1976), and included regular words (*n* = 49), exception words (*n* = 47) and pseudowords words (*n* = 30) presented in a random order, with a short practice session prior to the experimental trials. These words are matched for length and number of orthographic neighbors, but not frequency, with exception words being significantly higher in frequency than the regular words. A trial consisted of a fixation cross for 1500 ms followed by the experimental stimuli presented in uppercase, size 36 Times New Roman font. The word disappeared following 1500 ms or once a response was produced. Responses were produced into a Cyber Acoustics USB Stereo Headset and Boom Mic, and reaction times were measured from the onset of the stimulus to the triggering of the voice key. One participant had to be removed from the lexical decision study and one from the reading aloud study because of equipment malfunctioning.

For half of the participants, the behavioral session preceded the fMRI session, and for the other half the order was reversed. On average, the time between the behavioral and MRI session was 13.5 days, with a range from 0 (both sessions on the same day) to 48 days apart.

### fMRI Acquisition

A high-resolution T1-weighted structural and six task-based functional scans were acquired during an 80-min session using a 3T Siemens Magnetom Tim Trio scanner equipped with a 32-channel head coil. Scanning was done at the Core for Advanced Magnetic Resonance Imaging (CAMRI), at Baylor College of Medicine. A T1-weighted structural scan was collected first, followed by six consecutive 9.5-min functional scans. In between runs, subjects remained lying down in the scanner waiting for the next run to begin and were informed that the next run would begin shortly and to remain still. All 30 subjects participated in the imaging session. The T1-weighted structural scan involved the following parameters: TR = 2600 ms, TE = 3.03 ms, FoV = 256 mm, matrix size = 256 × 256, voxel size = 1 × 1 × 1 mm^3^. Functional runs were six 9.5-min scans obtained by using the following sequences: TR = 2000 ms, TE = 30 ms, FoV = 200 mm, voxel size = 2 × 2 × 2 mm^3^, slice thickness = 2 mm. A total of 280 volumes per run each with 62 slices were acquired in the axial plane to cover the whole brain. The task-based runs involved an ungrouped event-related design. Thirty (30) orthographic stimuli (20 familiar words, and 10 pronounceable pseudowords, Supplementary Material Appendix A) were presented once during each run, though participants were shown the same set of words across the 6 runs, with a different random order in each run. The repetition across runs was necessary for the RSA, as the approach compares the pattern of activation to individual words. However, we chose to cluster participants on the basis of the first run to avoid the effects of stimulus repetition on BOLD response. Stimuli were presented for 2 s, with a trial onset asynchrony of 12–20 s; this long ISI was selected on the basis of prior work with the clustering algorithm described below (Zhang et al., 2014; Zhao et al., 2017), and pilot data that suggested that the clustering algorithm was not well suited for rapid event related designs. Participants read the words aloud while they were in the scanner, and responses were recorded for accuracy, though data was not recorded in such a way that reaction times could be calculated.

### fMRI Data Analysis

Image preprocessing was conducted using SPM12 with the standard processing pipeline. The data were slice time corrected to correct for the difference in time between the first and the last slice acquired, realigned by removing motion artifacts, co-registered by overlaying the structural and functional images. For the clustering analysis and the univariate group-wise difference analysis, data was then normalized by warping to fit the standard the Montreal Neurological Institute (MNI) template, and spatially smoothed using a Gaussian kernel of FWHM 4 mm. For the RSA, no normalization or smoothing was carried out.

#### Clustering Analysis

Time series data from the first experimental runs was used for the clustering analysis. A 3D parcellation of the data were performed using the MarsBaR toolbox in SPM 12. The automatic anatomical labeling (AAL) brain atlas was used to obtain the parcellation, resulting in 90 ROIs, excluding the regions associated with the cerebellum. A Bayesian Nonparametric Spatio-Temporal Model for Multi-Subject fMRI Data was applied. This method has been reported previously in the literature (Zhang et al., 2014; Zhao et al., 2017). Methods are implemented in the Matlab GUI *NPBayes-fMRI* (Kook et al., 2017) processed data and software available at the GitHub address (https://github.com/rimehi/NPBayes_fmri, to be released publicly upon acceptance) that is comprised of two main interfaces, one for model fitting and one for the visualization of the results. Briefly, the clustering algorithm uses a unified, single stage Bayesian framework for the analysis of task-related brain activity in multi-subject fMRI data that eschews the traditional two-stage analysis which divides within subject analyses from between subject inferential statistics. Instead, the model specifically accounts for between-subject heterogeneity in BOLD response via a spatially informed multi-subject nonparametric variable selection prior. The model simultaneously estimates subject- and group-level statistical parametric maps of responses to different stimuli, with the subject level analysis borrowing strength in the estimation of the parameters from other subjects showing similar activation patterns. In this way, the model can cluster subjects into groups of individuals who show similar patterns of activation in response to specific kinds of stimuli. For model fitting, we used the default set of parameters provided in *NPBayes-fMRI*. Zhang et al. (2016) and Kook et al. (2017) describe the role of these parameters and offer general guidelines regarding the sensitivity of the results to the choice of their values. Zhang et al. (2016), in particular, show robustness of the results to the choice of several model parameters and only notice small sensitivity to the parameters that capture information on neighboring structure among ROIs. Here, we obtained a neighboring network by calculating Euclidean distances between pairs of ROIs using the coordinates defined in the MNI space and then thresholding the distances. We chose a threshold so that ROIs would have five neighbors on average. This information was used in the model to capture spatial correlation among neighboring ROIs when specifying an* a priori* probability of activation. Clustering of the subjects was consistent under small deviations of the prior specification from the default setting, with clustering configurations showing between 70% and 90% overlap. Activation maps were obtained by using a pre-specified Bayesian false discovery rate (BFDR) of 0.01, to control for multiple comparisons.

#### Between-Group Univariate Analysis

To ensure that the data used for clustering were independent of the validation data, preprocessed and normalized time series data from runs 2–6 were analyzed using a univariate approach. For the first-level analysis, data were entered into a subject specific, fixed-effect analysis using the general linear model, with one regressor based on the onset times for words and a second regressor based on the onset times for pseudowords (both modeled as events) deconvolved with a hemodynamic response function, along with six motion parameters and a regressor for scanner drift. For each subject, a *t*-map contrast of pseudowords vs. words was computed. These *t-maps* were then entered into a second level analysis that looked at whether the within subject pseudoword vs. word contrast differed as a function of subgroup assignment based on data from all stimuli from Run 1. Statistical thresholding was based on cluster-extent based thresholding, with a Gaussian Random Field cluster-size correction of family-wise error (FWE) *p* < 0.05. An initial conservative threshold analysis with the primary *p* < 0.001 and the cluster-size correction *k* = 42 failed to identify any clusters (Woo et al., 2014), so a more liberal analysis with the primary *p* < 0.005 and the cluster-size correction *k* = 95 was applied. Both of these correspond to a FWE *k-extent* threshold of *p* < 0.05. We then looked at the peak voxel of each cluster to determine the relationship between word and pseudoword activation for each subgroup to determine the source of the interaction.

#### Representational Similarity Analysis

For the RSA, no smoothing or normalization was applied during preprocessing. A general linear model predicting BOLD response that included the timing of each individual word (modeled as an event) deconvolved with a hemodynamic response function, six motion parameters and scanner drift was applied, resulting in beta-weights for each word in each run against fixation. We obtained 20 beta-weight maps for each subject, with each map reflecting the brain’s response to each word in the experiment. Following methods from previous studies (Rothlein and Rapp, 2014; Fischer-Baum et al., 2017), these beta-weight maps were then mean centered within each subject. Anatomical ROI analyses, based on warping the AAL map to each participant’s native space using the backward deformation fields generated from the SPM segmentation step, were applied to these 20 individual-word beta-weight maps for each participant. Specifically, we focused on four regions of interests (ROIs): the left IPL, the left AG, a broad ROI in the left ventral occipitotemporal lobe that combined the fusiform gyrus, inferior temporal gyrus and inferior occipital gyrus, and an IFG ROI that combined the pars triangularis and pars opercularis.

For each ROI and each participant, a vector of beta-weights for the voxels within that ROI was extracted for each of the 20 words, and a similarity matrix of word-to-word similarity for this region was calculated based on a tie-corrected Spearman correlation of the beta-weight vectors for each word to every other word. For each individual, the similarity between brain-based and theoretical similarity matrices was calculated. Phonological similarity was estimated using the phonological edit distance function in Phonological CorpusTools (Hall et al., 2016). In this measure, the similarity of two forms is calculated by calculating the number of one-feature changes needed to transform one phonological sting into another. Semantic similarity was estimated using an online pairwise latent semantic analysis (LSA) calculator^1^, a technique from distributional semantics that analyses meaning based on which words appear in similar contexts (Landauer et al., 1998). The result of the correlation between the brain-based and predicted similarity structure is taken to indicate the degree to which the region is processing phonological and/or semantic information in this group of participants.

Two analyses were carried out with this approach. First, we looked at the whole sample, asking whether the average correlation was significantly greater than zero. Second, we look at whether there are differences in these measures on the basis of which subgroup cluster they get assigned to, with a separate measure of semantic and phonological information processing for all ROIs calculated for each group.

## Results

### Overall Behavioral Results

The 30 participants in the study were all students at a highly competitive private university without a previous diagnosis of dyslexia. Table 1 reports descriptive statistics for the two standardized tests of reading skill. Unsurprisingly, their scores on standardized measures of reading skill are quite high. Based on the Nelson-Denny task, we found that they were in the 90th percentile for vocabulary knowledge, the 89th percentile for reading comprehension and the 72nd percentile for reading rate. Based on the Test of Word Reading Efficiency (TOWRE-2), they were in the 85th percentile for SWE and the 85th percentile for phonemic decoding efficiency.

**Table 1:** Descriptive statistics for standardized measures of reading skill.

		Raw score		Percentile	
		Mean	Range (IQR)	Mean	Range (IQR)
Nelson-Denny Form G	Reading comprehension	35.1/38	29–38 (2.8)	89th	35–99 (14)
	Vocabulary score	73.7/80	57–80 (5)	90th	51–99 (9.5)
	Reading rate	317 wpm	164–552 (146.3)	72nd	10–99 (36)
TOWRE-2	Sight word efficiency	103.1/108	80–108 (8)	85th	25–98 (21)
	Phonemic decoding efficiency	61.0/66	49–66 (4.8)	85th	47–98 (15.5)

Table 2 reports the group average results of the pseudohomophone lexical decision and the reading aloud study. We replicated the standard finding in the literature, with participants taking 13 ms longer to reject the pseudohomophones than matched pseudowords (*t*_(28)_ = 3.96, *p* = 0.0004^2^). We also replicated classic reading aloud effects of both lexicality and regularity. Participants took 87 ms longer to name pseudowords than words (*t*_(28)_ = 9.32, *p* < 0.0001) and were 23 ms slower with irregular words than with regular words (*t*_(28)_ = 3.88, *p* = 0.0005). Correlations across participants between these behavioral measures are reported in Table 3.

**Table 2:** Mean reaction times (RTs) in milliseconds with standard deviations in parentheses for the pseudohomophone lexical decision task and the reading aloud task.

		RT (SD)
Lexical decision	Words	588 (61.2)
	Pseudohomophones	611 (74.1)
	Matched pseudowords	598 (73.6)
Reading aloud	Regular words	492 (55.9)
	Irregular words	515 (62.3)
	Pseudowords	591 (102.4)

**Table 3:** Correlations between different behavioral reading measures.

	Reading comp.	Vocab. score	Reading rate	SWE	PDE	Pseudo homophone	Lexicality	Regularity
Reading comprehension	1.0							
Vocabulary score	0.33^†^	1.0						
Reading rate	0.26	0.07	1.0					
Sight word efficiency (SWE)	0.03	−0.22	0.42*	1.0				
Phonemic decoding efficiency (PDE)	0.59***	0.03	0.50**	0.39*	1.0			
Pseudohomophone effect	0.27	0.15	0.12	0.21	0.25	1.0		
Lexicality	0.17	0.07	0.21	0.02	0.33^†^	0.06	1.0	
Regularity	0.00	0.03	−0.19	−0.07	0.08	0.04	0.51**	1.0

^†^p < 0.1, *p < 0.05, **p < 0.01, ***p < 0.001.

The critical test in this study is whether there were between group differences on these effects (pseudohomophone, lexicality and regularity), when groups are defined based on clustering of the fMRI data. In the next section, we describe the results of the fMRI clustering algorithm, and subsequent fMRI analyses based on these clusters. Finally, we reanalyze the behavioral data on the basis of these subgroups.

### fMRI Results

#### Behavioral Results

Overall, participants were highly accurate in reading words and pseudowords aloud in the scanner (mean = 98.2% correct, SD = 3.1%). All participants participated in six runs of the task, with the same set of 30 words repeated in each run. Three of the participants made a large number of no response errors (>10) in a single run. The first reported that she forgot the instructions, the second that she dozed off for part of one run and the third that his glasses needed to be adjusted. These runs were excluded from subsequent analysis. Participants made the occasional error in decoding nonwords (<0.5% of all nonword trials), and there was only a single instance of a word being pronounced incorrectly.

#### Clustering Analysis

**Figure 1:**
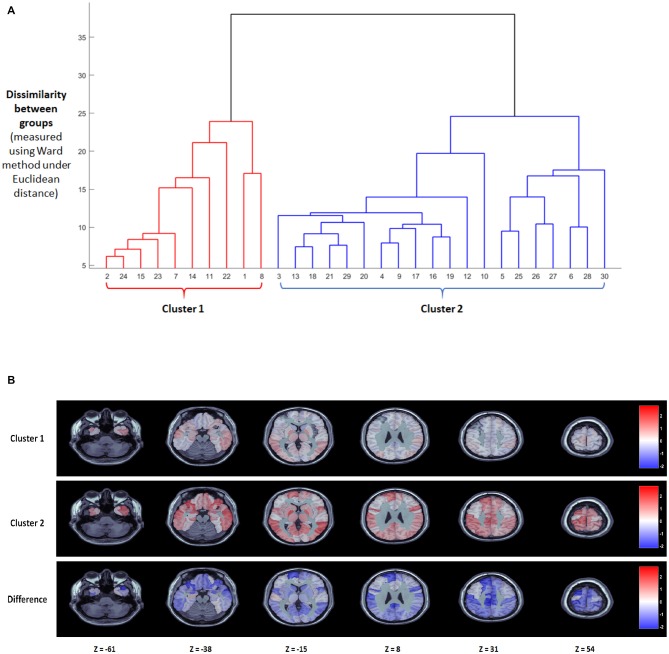
Results of the nonparametric Bayesian model analysis treating all words as a single stimulus type. **(A)** Cluster dendrogram obtained with hierarchical cluster under the linkage method. Two clear clusters of subjects emerge with a single subject (10) appearing as an outlier. **(B)** Posterior group-level maps of β for the two largest clusters at six axial slices (top two rows) as well as the difference between the two clusters (bottom).

The results of the model estimation are shown in Figure 1, based on a comparison of all words to a blank screen baseline, with BFDR = 0.01, to control for multiple comparisons. The top of Figure 1 shows the cluster dendrogram indicating which subjects show a similar pattern of response to written words based on AAL regions. Based on the dendrogram obtained via hierarchical clustering on the thresholded beta coefficients, we select two as number of clusters. Measures commonly used for interpretation and validation of consistency within clusters of data, such as the within cluster sums of squares and the average silhouette, returned two as the optimal number of clusters with 10 subjects in Subgroup 1 and 20 subjects in Subgroup 2.

The bottom of Figure 1 shows the posterior group-level β-maps for these two subgroups at six axial slices, as well as the difference in the βs between the two subgroups. The Bayesian framework of the clustering algorithm means that it would be inappropriate to do traditional frequentist statistics to determine which regions differ significantly across groups. However, we can determine which AAL regions show large β-weights (>1) in each group, and which AAL regions have a numerical difference in *a posteriori* β-weights response across groups. These regions are compared to the Taylor et al. (2013) meta-analysis neuroimaging findings of the neural substrates of reading aloud.

Both subgroups showed increased activation to words over baseline in bilateral occipitotemporal regions involved in visual processing of written words (inferior Occipital Sulcus, Lingual Gyrus, as well as the Left Fusiform Gyrus), and bilaterally in the superior temporal lobe (Superior Temporal Gyrus, Heschl’s Gyrus), which is involved in phonological processing. Most notably, the two groups appear to differ by degree rather than by double dissociation. The second subgroup showed higher β values than the first subgroup across the cortex. Regions that had high β-values for the second subgroup and low β-values for the first subgroup included left lateralized parietal regions (Supramarginal Gyrus, IPL). Notably, this included regions that have been argued to be involved in mapping from letters to sounds. The second subgroup also showed activation in temporal regions where the first subgroup did not, specifically the superior temporal pole, which has been argued to be involved in semantic storage, and bilateral frontal regions (IFG pars triangularis and opercularis, insula, precentral gyrus), some of which have been implicated in semantic access and others which have been implicated in the articulatory aspect of spoken production. Finally, the second subgroup had large β-values in a number of subcortical regions (Thalamus, Pallidum, Caudate Nucleus, Midcingulate Gyrus) where the first subgroup did not. Overall, there were significant differences in accuracy for the scanner task between Group 1 and Group 2. Group 1 made significantly more errors (3.6%) than Group 2 (0.8%, *t*_(28)_ = 2.56, *p* = 0.016).

#### Between-Group Univariate Analysis

The second fMRI analysis was a whole brain comparison looking at how an individual’s response to word vs. pseudoword stimuli. First, we looked at an analysis that collapsed across subgroups, to ensure that the findings of the current experiment matched previous studies. With a primary *p* < 0.001 and a Gaussian Random Field theory cluster-size correction to a FWE of *p* < 0.05, we found 10 clusters that showed greater activation for words than for nonwords and 12 clusters that showed greater activation for nonwords than for words. Figures and tables with the details of the results of these analyses are in the Supplementary Materials (Supplementary Figure S1, Supplementary Tables S1, S2).

Briefly, for the words greater than nonwords contrast, we found a large cluster (>500 voxels) in the left AG and middle occipital temporal cortex, consistent with what has been previously reported in the literature (Binder et al., 2009). We also found moderately sized cluster (100–500 voxels) in the primary visual cortex, a cluster that is largely contained within the anterior portions of the left fusiform gyrus, and a right middle temporal gyrus cluster. There was also a moderately sized cluster in a medial parietal region in the left precuneus, with a smaller cluster (<100 voxels) in the homologous right hemisphere region. Finally, smaller clusters were found in the right supramarginal gyrus and three smaller right hemisphere clusters in the visual cortex (calcarine, lingual gyrus and superior occipital gyrus).

For the nonwords greater the word contrast, there were extremely large clusters in the both left (2706 voxels) and right (1253 voxels) frontal cortex, centering on the IFG and the anterior insula cortex. There was also a large bilateral cluster (856 voxels) in the left and right medial aspect of the superior frontal gyrus. Two sizeable clusters were found in the left IPL, consistent with the findings of the Taylor et al. (2013) meta-analysis. The remaining clusters included two smaller right orbital frontal clusters, a larger right cerebellar cluster, a larger cluster in the right AG and several subcortical clusters, including the left caudate and the right thalamus.

**Figure 2:**
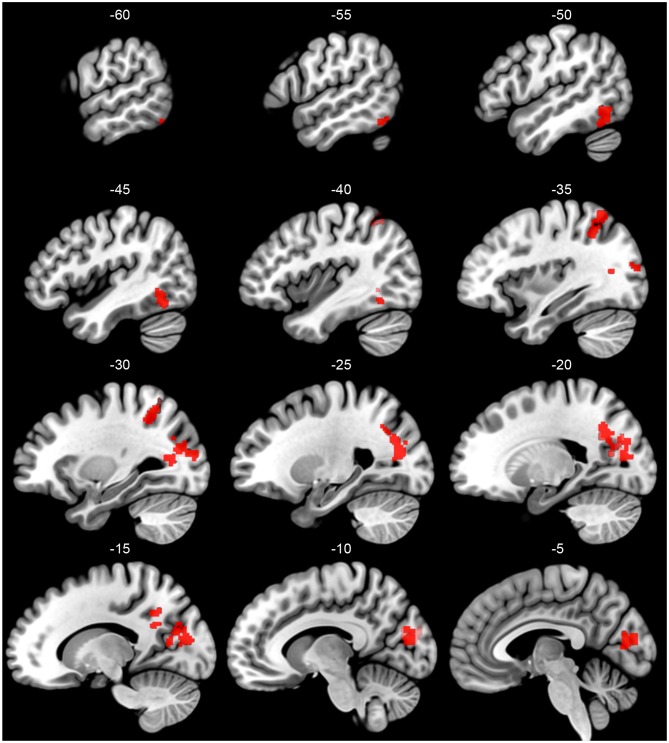
Sagittal slices of the left hemisphere regions that show a significant between-group interaction in the pseudoword vs. word comparison (primary *p* < 0.005, cluster-size threshold p_FWE_ < 0.05). Group is identified based on a clustering of participants on their BOLD responses to all word and pseudoword stimuli in the first run of the experiment. Pseudoword vs. word contrast carried out over runs 2–6.

Critical for the current study, we next investigate how these patterns of activation varied as a function of subgroup assignment. With a primary *p* < 0.005 and a Gaussian Random Field theory cluster-size correction to a FWE of *p* < 0.05, or a cluster extent threshold of *k* = 95, four significant clusters emerged showing this interaction, shown in Figure 2 and reported in Table 4. The largest cluster includes 735 voxels bilaterally in the occipital cortex, superior to the calcarine fissure and extended up in the cuneus gyrus. Looking at the peak voxel in this cluster, individuals in Subgroup 1 show a greater activation for words than for pseudowords, while individuals in Subgroup 2 show a greater activation for pseudowords than for words. Another cluster of 339 voxels falls largely in the right precuneus and right angular gryus. Like the primary visual area cluster, at the peak voxel in this cluster, Subgroup 1 shows greater activation for words than for pseudowords, while Subgroup 2 shows greater activation for pseudowords than for words. A third cluster of 132 voxels falls primarily in the left IPL, though extends slightly in the superior parietal lobule. Unlike the first two clusters, in this region both Subgroup 1 and Subgroup 2 show greater activation to pseudowords than to words, but Subgroup 2 shows a much larger effect. Critically, this IPL region aligns very closely with the region identified by the Taylor et al. (2013) meta-analysis as the region most likely to be involved in sublexical processing. Finally, a cluster of 115 voxels falls near the canonical visual word form area (VWFA) at the junction of the left inferior occipital and temporal cortex. As with the IPL cluster, in the VWFA cluster, subgroup 2 showed greater activation for pseudowords than for words, while subgroup 1 showed equivalent activation for both types of orthographic stimuli. The Taylor et al. (2013) meta-analysis also identified this region as showing greater activation for pseudowords than for words. There were no regions in which Group 1 showed a greater difference between pseudowords and words than Group 2.

**Table 4:** Significant clusters of between-group interaction of pseudoword vs. word contrasts (primary *p* < 0.005, Gaussian Random Field cluster-size threshold *p*_FWE_ < 0.05), including the MNI coordinate of the peak voxel and at most two other local maxima (>8 mm distance).

Clusters	Cluster size	MNI coordinates	Subgroup 1	Subgroup 2
		[x, y, z]		
Right precuneus/	735	[16, −64, 40]	Words >	Pseudo >
Angular gyrus		*[32, −56, 40]*	Pseudo	Words
		*[38, −60, 50]*
Bilateral primary	303	[−12, −82, 13]	Words >	Pseudo >
visual cortex		*[−24, −74, 14]*	Pseudo	Words
		*[14, −68, 16]*
Left inferior	132	[−32, −52, 48]	Words =	Pseudo ≫
parietal lobule		*[−36, −58, 58]*	Pseudo	Words
Visual word	115	[−46, −60, −12]	Words =	Pseudo ≫
form area		*[−52, −60, −20]*	Pseudo	Words
		*[−60, −60, 18]*

*Relation of word to pseudoword activation for each subgroup at the peak voxel is also reported*.

#### Representational Similarity Analysis

**Figure 3:**
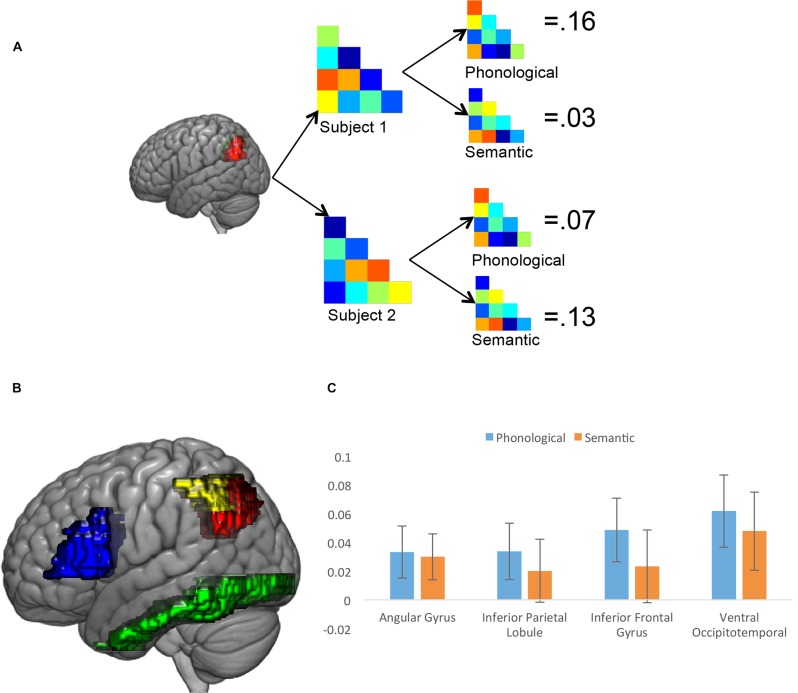
Results of the representational similarity analysis (RSA). **(A)** Illustration of the analysis approach, calculating the brain-based correlation for each participant in each region of interest (ROI), and then comparing the brain-based correlation with the semantic and phonological similarity measures. **(B)** ROIs used in the RSA analysis. Red is left angular gyrus (AG), yellow is left inferior parietal lobule (IPL), blue is left inferior frontal gyrus (IFG) and green is the left ventral occipitotemporal cortex (vOT) **(C)** Results of the whole-group analysis for four ROIs. Error bars reflect ±1 SEM.

A final fMRI analysis used RSA based on the 20 familiar words used in the experiment to map the phonological and semantic processing of different anatomical ROIs, defined based on the Taylor et al. (2013) meta-analysis. Specifically, we looked at semantic and phonological processing in the left AG and the left IPL, the left IFG and the ventral occipitotemporal cortex (vOT). The result of the analysis that looks at all participants, ignoring subgroup membership, is shown in Figure 3. In all four of the ROIs, the correlation between the brain-based similarity matrix and the predicted phonological similarity matrix was statistically significantly greater than 0, using a one-tailed *t*-test (AG = 0.033, *t*_(29)_ = 1.83, *p* = 0.039; IPL = 0.034, *t*_(29)_ = 1.72, *p* = 0.048; IFG = 0.049, *t*_(29)_ = 2.20, *p* = 0.018; vOT = 0.062, *t*_(29)_ = 2.45, *p* = 0.010). The correlation between the group-average brain-based similarity matrix and the predicted semantic similarity matrix was statistically significant in the vOT region (0.048, *t*_(29)_ = 1.76, *p* = 0.045) and in the AG region (0.03, *t*_(29)_ = 1.76, *p* = 0.036). These results show that, at least in the context of a reading aloud task, there is clear phonological processing of written words in frontal speech production areas, the AG, the IPL and the vOT, and semantic processing in the vOT and the AG.

One particularly surprising aspect of these results is that the pattern of activation in the AG is correlated with both phonological and semantic processing. The AG has been identified as a part of the brain’s semantic network (e.g., Binder et al., 2009), largely due to the fact that it shows greater activation to words than to pseudowords, as we show in our own results above and as Taylor et al. (2013) find in their meta-analysis, and because its activity correlates with word frequency and to a lesser extent imageability (Graves et al., 2009). However, as Taylor et al. (2013) point out, these results are consistent with either a region that processes lexical phonological information or a semantic region, the current results suggest that this region is engaged with both of these functions during in reading aloud, though previous results from our lab (Fischer-Baum et al., 2017) reported orthographic processing in this region during a different reading task. An alternative interpretation is that there is variation across anatomical atlases for what is considered part of the AG (Seghier, 2013); perhaps in the broader IPL there are regions that are doing semantic processing of words and other regions that are doing phonological processing, and the precise boundaries between these regions do not fall clearly on anatomical divisions. The region that the AAL brain atlas identifies as being the AG may, for example, include what other atlases might identify as supramarginal gyrus, which has been identified as playing a phonological role in reading (e.g., Stoeckel et al., 2009). The finding that our AG region is both phonological and semantic may have more to do with an inconsistent use of brain labels in the literature, rather than a finding that is inconsistent with previous claims.

Critical for current study, though, is whether there are differences in these RSA results as a function of subgroup assignment from the clustering analysis. For each ROI, subgroups were compared on the phonological and semantic similarity measures. Subgroup 2 had a significantly higher phonological similarity index in all four ROIs (AG: −0.02 vs. 0.06, *t*_(28)_ = 2.24, *p* = 0.033; IFG: −0.01 vs. 0.08, *t*_(28)_ = 2.11, *p* = 0.049; IPL: −0.03 vs. 0.07, *t*_(28)_ = 2.46, *p* = 0.020; vOT: −0.02 vs. 0.10, *t*_(28)_ = 2.51, *p* = 0.018). Strikingly, the vOT did not show between group differences in semantics (Subgroup 1 = 0.02, Subgroup 2 = 0.06, *t*_(28)_ = 0.80, *p* = 0.43), and neither did any of the other ROIs (AG: −0.01 vs. 0.05, *t*_(28)_ = 1.93, *p* = 0.063; IFG: −0.01 vs. 0.04, *t*_(28)_ = 0.85, *p* = 0.404; IPL: −0.02 vs. 0.04, *t*_(28)_ = 1.45, *p* = 0.158).

Subgroups identified by the clustering analysis differ with respect to phonological processing, with Subgroup 2 showing higher correlations with the phonological similarity matrix than Subgroup 1 in all four ROIs. However, the two groups do not differ in semantic processing of written words. It is worth noting that Subgroup 1 generally performed worse on the task in the scanner, meaning that they were less accurate at generating the correct phonological form, consistent with the RSA results.

#### Behavioral Results by fMRI Subgrouping

Finally, we reanalyzed the behavioral data based on the groupings generated from the fMRI data. We were primarily interested in between-group differences in the pseudohomophone, lexicality and regularity effects but we also tested whether the groups differed on standardized measures of reading skill.

**Figure 4:**
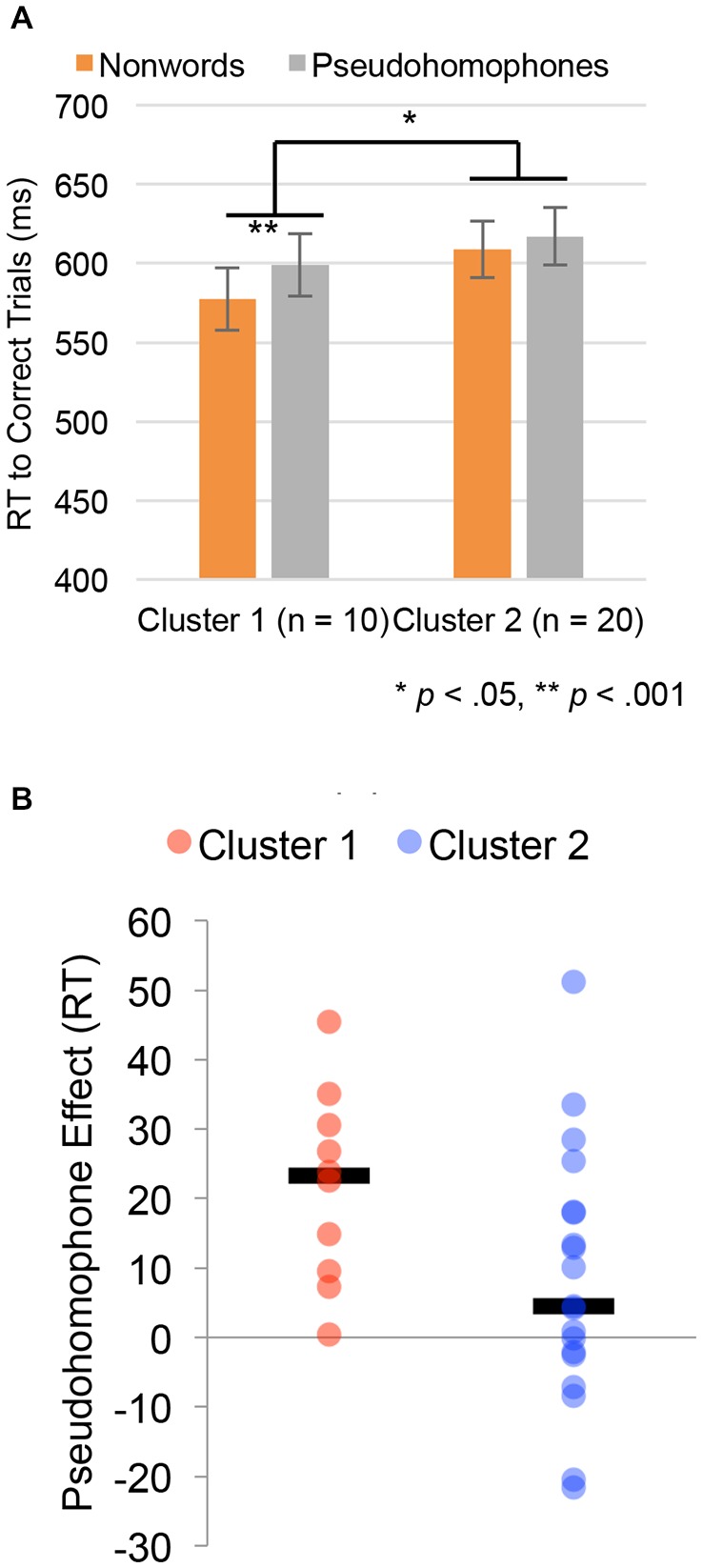
Behavioral results in the pseudohomophone effect lexical decision experiment treating cluster assignment from the single stimulus type analysis as a between group variable. **(A)** Group differences in reaction times (RTs) for the pseudohomophone and other pseudoword stimuli. Error bars are ±1 SEM. **(B)** Plot of the median pseudohomophone effect (black bar) and individual subject data for individuals in each cluster.

The pseudohomophone experiment was reanalyzed using subgroup-membership as a between-subject factor. The results of this analysis are shown in Figure 4. Figure 4A shows reaction time for correct trials in the pseudohomophone lexical decision task, for both pseudohomophones and other nonwords, by subgroup. There were no significant differences between subgroups 1 and 2 in the overall lexical decision time (Subgroup 1 = 587 ms, Subgroup 2 = 606 ms, *t*_(27)_ = 0.77, *p* = 0.45). However, the group by nonword-type interaction was significant (*F*_(1,26)_ = 5.01, *p* = 0.034). Specifically, individuals in Subgroup 1 had a robust difference between pseudohomophones and other nonwords (599 vs. 577, *t*_(9)_ = 4.98, *p* = 0.0008), while individuals in Subgroup 2 showed a small, marginally significant (617 vs. 608, *t*_(19)_ = 2.15, *p* = 0.060). These differences can be seen at the individual subject level. Figure 4B plots individual subject data of the size of the pseudohomophone effect for the two clusters, with median values indicated by a black line.

**Figure 5:**
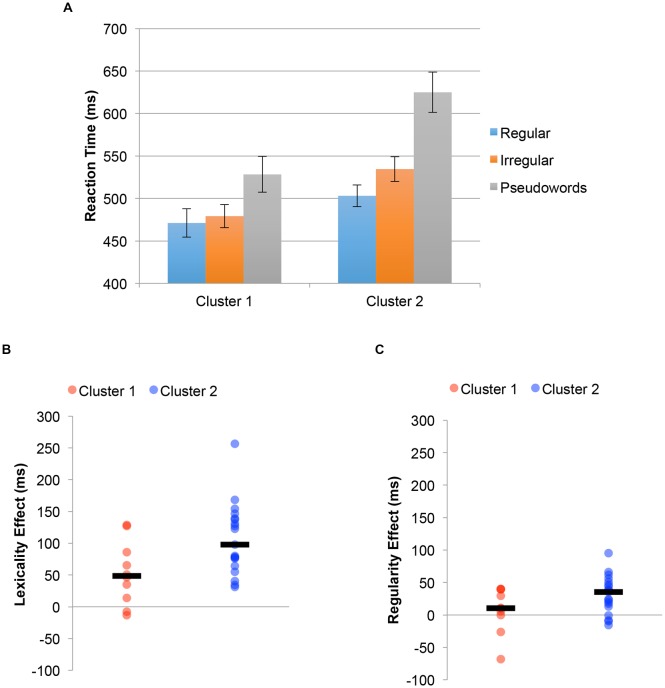
Behavioral results in the reading aloud regular, irregular and pseudowords experiment defining groups based on the clustering from functional magnetic resonance imaging (fMRI) data. **(A)** Group differences in RTs for the pseudohomophone and other pseudoword stimuli. Error bars reflect ±1 SEM. **(B)** Plot of the median lexicality effect (black bar) and individual subject data for individuals in each cluster. **(C)** Plot of the median regularity effect (black bar) and individual subject data for individuals in each cluster.

The reading aloud experiment was also reanalyzed using cluster-membership as a between-subject factor. The results of this analysis are shown in Figure 5. Figure 5A shows reaction time for regular words, irregular words and pseudoword reading time. There was a significant difference between groups 1 and 2 in reading time (Subgroup 1 = 493 ms, Subgroup 2 = 554 ms, *t*_(27)_ = 2.46, *p* = 0.020), with subgroup 1 being faster will all types of stimuli (Regular Words = 32 ms; Irregular Words = 55 ms; Pseudowords = 97 ms). Still, the group by stimulus-type interaction was significant (*F*_(2,54)_ = 4.94, *p* = 0.011). Individuals in Subgroup 2 showed a larger lexicality effect (all words vs. pseudowords: 106 ms) than individuals in Subgroup 1 (53 ms, *t*_(27)_ = 2.40, *p* = 0.023), and also a trend in the direction of a larger regularity effect (irregular words vs. regular words; Subgroup 1: 8 ms, Subgroup 2: 31 ms, *t*_(27)_ = 1.71, *p* = 0.099). Again, the lexicality differences can be seen at the individual subject level, with Figure 5B plots individual subject data of the size of the lexicality effect for the two clusters, with median values indicated by a black line. Figure 5C plots the same analysis for the regularity effect. For the simple main effects, Subgroup 2 was significantly slower than Subgroup 1 for exception words (535 ms vs. 479 ms, *t*_(27)_ = 2.46, *p* = 0.021) and nonwords (625 ms vs. 529 ms, *t*_(27)_ = 2.77, *p* = 0.010). While there was a trend towards being slower in regular word reaction time as well, the difference was not significant (503 ms vs. 471 ms, *t*_(27)_ = 1.52, *p* = 0.14).

Finally, Table 5 reports between group differences on standardized reading measures. Subjects from the different subgroups did not differ significantly on any of the standardized reading measures, indicating that these other behavioral and neuroimaging differences did not correspond to differences in reading skill. One particularly surprising result from this analysis is the failure to find group differences on the PDE task, which is typically used to assess sublexical reading ability. One would expect scores on this task to be positively correlated with the other indices of sublexical reading, like the pseudohomophone effect and the regularity effect. As can be seen in Table 3, there is no correlation. One might also expect the subgroups to differ on the PDE task, if they differ on sublexical efficiency, but as can be seen in Table 5 there are no differences here either. One possible explanation for this is that nearly all of our participants performed near ceiling on this task. The phonemic decoding efficiency task might not be as sensitive to subtle differences in sublexical processing between highly literate readers as the other indices discussed above.

**Table 5:** Differences in standardized reading scores by group.

	Cluster 1 (*n* = 10)	Cluster 2 (*n* = 20)
Reading comprehension	93.7%	91.6%
Vocabulary score	94.0%	91.2%
Reading rate	317	317
Sight word efficiency	101.5	103.9
Phonemic decoding efficiency	61.3	60.8

## Discussion

Even within a range restricted set of highly competent, adult, literate readers, individuals vary in their performance on reading tasks. This variability was observed in high-level measures of reading skill like the ability to comprehend passages and in more specific, cognitively informed measures, like the time it takes them to reject pseudowords that are pronounced like real words in a lexical decision task or the time it takes to read nonwords. Furthermore, these differences in reading behavior are linked to differences in how the brain responds to written words. Using a novel, data-driven approach to analyzing multi-subject, task-based fMRI data, two subgroups of readers were identified on the basis of how their brains response to words and pseudowords in a reading aloud task.

Follow up neuroimaging analyses with an orthogonal set of data suggest that these subgroups differed in phonological processing of written stimuli. First, the size of the pseudowords > words contrast was significantly larger for the second subgroup than the first subgroup in several regions of the brain, most notably in the left IPL and the left vOT. Second, the extent to which the similarity relationships among the fine-grained patterns of activation to specific words matched the phonological similarity between the words was greater in the second subgroup than the first subgroup in the left IFG, the left AG, and the left IPL. Finally, the first group showed a larger pseudohomophone effect, that is they were slower at rejecting pseudowords that sound like real words (e.g., BRANE) than rejecting matched pseudowords (e.g., BRAME), than the second group, while the second group showed a larger lexicality effect, that is they were slower at reading pseudoword (e.g., PENK) than word stimuli (e.g., YARN), than the first group. Taken together, one interpretation of this finding is that participants in the first cluster have more efficient and less effortful sublexical processing of words than individuals in the second cluster.

Our findings are particularly noteworthy in that the data used to cluster participants was fully independent of the data used to validate that clustering. The behavioral data that was collected to validate the fMRI clustering was collected during a different testing session, on average 2 weeks from the fMRI session, and included a variety of reading tasks, both reading aloud and lexical decision. As discussed above, previous research has shown that the reading system changes strategically as a function of task demands. Based on this prior research, we assume that participants in the current experiment are varying their lexical and sublexical reading routes based on task-demands during both the imaging task in the scanner and the behavioral tasks outside of the scanner. Despite that, the clustering algorithm applied to the neuroimaging task is picking up on some individual variability that appears to be stable across time and task.

What then is this stable difference between readers that is being identified in these experiments? Both behavioral and neuroimaging analyses suggest that the clustering algorithm has identified readers who differ in their sublexical reading route. Behaviorally, the group that has the larger lexicality effect also shows a significantly smaller pseudohomophone effect. The pseudohomophone effect has largely been interpreted to reflect the fact that when we are presented with pronounceable written stimuli, we automatically generate a phonological representation, even during tasks that do not require overt pronunciation like visual word recognition, which in turn influence other processes associated with word reading, like the activation of semantics (Van Orden, 1991; Frost, 1998; Harm and Seidenberg, 2004; Braun et al., 2009; Leinenger, 2014)^3^. This phonological activation poses a particular challenge in lexical decision which requires only knowledge of word spelling, with many theories proposing an additional “spelling check” step by which the brain compares a spelling predicted by activated phonology and semantics with the actual stimuli. Groups could differ in the size of the pseudohomophone effect either because they differ in the amount that phonological information is competing with orthographic information when making the lexical decision or they could differ in the “spelling check” mechanism. As we discuss below, both of these group differences could contribute to the observed findings.

One possibility is that readers in Subgroup 1 have a greater degree of competition between orthographic and phonological representations than readers in Subgroup 2. The degree of competition could be driven, in part, by the speed of the sublexical reading route relative to the lexical route. Subgroup 1 is generally faster at reading aloud than Subgroup 2, but the difference is more pronounced for nonwords (97 ms difference) than exception words (55 ms difference) or regular words (37 ms difference). The readers who show a smaller pseudohomophone effects are also more delayed at generating pronunciations from pseudoword stimuli relative to the speed with which they generate pronunciations for word stimuli, perhaps because the use of the sublexical route is more effortful for this group. As a result, information from those pronunciations is less likely to interfere in the lexical decision task.

Some of the results of the neuroimaging analyses also support this difference in processing between the two groups. Both groups show greater activation for pseudowords than words in a large cluster in the IPL, a region identified as being critical for sublexical processing during reading aloud (Taylor et al., 2013), but Subgroup 2 shows a greater difference in activation between the two types of stimuli than Subgroup 1. One way to interpret this difference is to assume that the groups differ in the effort that it takes to use the sublexical route. Taylor et al. (2013) propose a u-shaped BOLD response function on the basis of engagement and effort, with a lack of engagement showing the smallest BOLD response, effortful engagement showing the largest BOLD response, a greater response than less effortful engagement. If we assume that this region is engaged in the mapping from letters to sounds in both groups, then the difference between Subgroup 2 and Subgroup 1 is that this process for the same set of pseudowords is more effortful for Subgroup 2, which is consistent with the fact that Subgroup 2 is slower at pseudoword reading than Subgroup 1.

Subgroup 2 also shows more pseudoword than word activation that Subgroup 1 in other cortical regions, for example in the VWFA, a region that has been postulated to represent sublexical orthographic form (Dehaene et al., 2005; Fischer-Baum et al., 2017). In reading aloud studies, it is typical for this region to show greater activation for pseudowords than for words (Taylor et al., 2013), though equivalent word and pseudoword activity is often reported in passive tasks that do not require overt naming (e.g., Cohen et al., 2002). Taylor et al. (2014) interpreted this difference as reflecting top-down signals from regions that are generating phonological information from unfamiliar words (see also Price and Devlin, 2011). These proposed top-down signals operate like the “spelling check” function described above; that is, the top-down signal is generated the prediction of the word’s spelling and there is greater activation when there is a mismatch between the stimuli and the prediction. Under this interpretation, individuals in Subgroup 2 are using this top-down signal more than individuals in Subgroup 1. Note that this result is in the context of a reading aloud experiment without pseudohomophones. Therefore, the between group difference in the pseudohomophone effect could be explained by a difference in the reliance on the top-down “spelling check” signal.

In addition to looking for differences in univariate activation, we looked for between group differences in information processing using RSA. There were no differences between the groups in the degree of semantic information elicited by the written words. The groups did show differences in phonological information processing in the left vOT, IPL, the AG, and the IFG. Specifically, Subgroup 2, which showed both behavioral and univariate neuroimaging evidence for more effortful sublexical processing, showed higher correlations between the brain-based similarity structure in those regions and the predicted similarity structure based on phonology. On the surface, this result may appear to go in the opposite direction of what would be predicted by the group for whom sublexical processing is less effortful. However, given that the task in the scanner is a reading aloud task, it is clear that both groups are activating phonological representations of the written words. Some of the words being read aloud in the scanner had irregular spelling-to-sound correspondences (e.g., YACHT). The phonological similarity matrix is based only a word’s received pronunciation (e.g.,/jat/), which is generated by the lexical reading route. For these irregular words, the sublexical reading route may be activating alternative pronunciations of the written word (e.g.,/jækt/,/jætft/,/jæt/, etc.) which compete with the received pronunciation. Readers in Subgroup 2, whose sublexical processing requires more effort, will be activating the received pronunciation more quickly than these alternative pronunciations. As a result, for Subgroup 2, the phonological representation in these regions is closer to the received pronunciation and therefore correlates higher with the phonological similarity matrix. For Subgroup 1, the phonological representation in these regions will be farther from the received pronunciation and therefore correlate less with the phonological similarity matrix. This interpretation is particularly compelling if we assume that the AG plays a role in processing lexical phonological information (Taylor et al., 2013), and therefore its activation reflects selection of the received pronunciation rather than the generation of a possible pronunciation by the sublexical route. While not specifically designed to contrast regular and irregular words, it is worth noting that many of the word stimuli in the in-scanner task were irregular (e.g., YACHT, EYE, SEW). An additional prediction of this account would be that differences in phonological similarity between Subgroup 1 and Subgroup 2 in these regions would be driven by the irregular words only. However, the current experiment is underpowered to examine this prediction.

This interpretation is challenged a little by the observation that readers in the two subgroups differ in ways beyond our indices of sublexical processing. In the scanner, readers in Subgroup 1 performed more poorly, and outside of the scanner, they were significantly faster overall in the reading aloud study and numerically faster in the lexical decision study. Therefore, these subgroups may differ on overall processing speed or perhaps on where they place the criterion in a speed/accuracy tradeoff. Faster reading time could explain the fact that the difference between the two groups identified by the clustering algorithm was one of degree rather than dissociation. Longer reaction times might result in larger BOLD responses, meaning that the algorithm might have clustered on the basis of faster vs. slower readers rather than differences in reading strategies (Binder et al., 2005). While this difference may explain some of our results, we think that this interpretation is unlikely the only account for all of our findings. The standardized reading batteries should be sensitive to differences in overall processing speed or in speed/accuracy tradeoff, but no differences were observed. Furthermore, it is unclear how this account could explain the behavioral interactions, with Subgroup 1 showing a particularly large pseudohomophone effect in reaction time and Subgroup 2 showing even greater slowing for pseudowords compared to regular word stimuli.

We conclude that our current study suggests individual differences in how effortful engagement of the sublexical pathway is in highly literate adult readers. We can only speculate as to the source of this variability. Woollams et al. (2016) suggest that developing readers come into the learning process with differences in phonological processing abilities. Children with stronger phonological representations are more likely to learn to read through a sublexical dominant pathway, while children with weaker phonological representations will tend to rely on the lexical pathway. These differences in the initial stages of learning are still reflected in how words are processed in the adult brain. While we did not collect any measures of phonological processing ability in the current study, Luque et al. (2011) showed that the size of pseudohomophone effect varied with individual’s categorical perception ability, with those individuals with worse phonological processing ability also showing a reduced pseudohomophone effect (see also Holyk and Pexman, 2004). This finding is consistent with the proposal laid out above. If we assume that adults with more effortful phonological processing from written words were also children with weaker phonological processing ability, then learning to read with weaker phonological representations results in a slower, more effortful sublexical/phonological route, which in turn diminishes the pseudohomophone effect.

Strikingly, this difference between readers does not appear to map onto any differences in reading skill, suggesting that there are multiple reading modes that can be equally effective organizations of the reading network. These findings run contrary to claims of the stage-based theory of reading development that assumes that young readers first develop their sublexical route, and later develop more the efficient lexical route (Ehri, 1992; Share, 1995). Indeed, as children learn to read better the size of the pseudohomophone effect is reduced (Sprenger-Charolles et al., 2003; Grainger et al., 2012) indicating less of a reliance on the sublexical/phonological route. Extending this claim to adult readers, we would predict that relying more on sublexical/phonological route, and therefore showing a greater pseudohomophone effect, would be related to worse overall reading skill. However, we find no differences between the two clusters—defined by fMRI but also differing on pseudohomophone and lexicality effect size—in terms of standard measures of reading performance in either the TOWRE-2 or the Nelson-Denny Test.

Taken together, our results suggest that there is heterogeneity even within a group of highly-skilled readers. This heterogeneity is detectable in behavioral measures of reading ability and it is detectable in the brain’s response to written words. Heterogeneity of this type has important implications for our understanding of reading in the brain. For example, much of the research into developmental dyslexia asks how reading disordered individuals, as a group, deviate from neurotypical readers, as a group, in both behavioral and neuroimaging measures (Ramus, 2003; Richlan, 2012). Serious concerns have been raised about treating individuals with dyslexia as a homogenous group (Hadzibeganovic et al., 2010) as a range of different underlying impairments, with corresponding differences in brain structure and function, could lead to reading disabilities. A similar concern could be raised for treating neurotypical readers as a homogenous group, as it is clear from the current study that individuals vary in how their brain responds to written words. Based on our current study it seems like there are two subtypes of readers. It is premature to draw conclusions about the precise prevalence of reading subtypes in the general population from the sample used in our experiment. However, it is unlikely that differences in reading style would be classified as what de Schotten and Shallice (2017) call a “minority-discrepant situation”, that is a situation in which most individuals show one pattern with only a few outlying subjects. Instead, this difference appears to be a “major-discrepancy situation”, with multiple subtypes of readers frequently observed in the population. As de Schotten and Shallice (2017) discuss, major discrepant situations have the largest consequences for drawing inferences from group-average data.

The individual differences in brain activation reported in the current study likely reflects only a fraction of the heterogeneity that exists in literate adults. Welcome and Joanisse (2012) also found that activation to other cortical regions (e.g., precuneus, middle temporal gyrus) correlate with a measure of lexical reading ability (SWE), while we found no differences between our subgroups on lexical measures. One limitation of the clustering approach taken here is that readers may differ on a number of dimensions. Our analysis may have picked up on the primary dimension that distinguishes readers in our sample—the effort needed to use the sublexical reading route—but readers in our sample may also differ on the effort needed to is the lexical pathway as well. Furthermore, activation patterns are not the only source of heterogeneity in the reading brain that could be related to individual differences in reading behavior. Structural differences in white matter connectivity have also been linked to differences in sublexical reading ability (Welcome and Joanisse, 2014) and semantic processing when reading (Graves et al., 2014). In general, understanding the ways that adult, neurotypical readers vary both in their reading behavior and their reading brains provide clear benchmarks that will allow existing models of reading in the brain to extend to explaining individual differences.

## Author Contributions

SF-B and MV designed the study and interpreted the results. SF-B wrote the initial draft of the manuscript, though all authors contributed to subsequent draft. JHK designed and implemented the novel fMRI statistical analysis. YL performed the behavioral experiments and analyzed the data. AR-N performed the neuroimaging experiments and preprocessed the neuroimaging data.

## Conflict of Interest Statement

The authors declare that the research was conducted in the absence of any commercial or financial relationships that could be construed as a potential conflict of interest.

## Footnotes

^1^lsa.colorado.edu/

^2^Statistical tests for reaction time data were carried out after a log transformation.

^3^In addition to this phonological explanation, Harm and Seidenberg (2004) suggest a limited contribution of direct mappings between orthography and semantics in the pseudohomophone effect. With appropriately matched nonword controls, as in the current study, these effects are very small and therefore we think that it is unlikely that these between group differences have to do with between group differences in orthography to semantics mapping.
