# Supplementary material for: Individual Differences in the Neural and Cognitive Mechanisms of Single Word Reading
Source: Front Hum Neurosci. 2018 Jul 5;12:271. doi: 10.3389/fnhum.2018.00271 (PMC6041384; doi:10.3389/fnhum.2018.00271)
Supplement: Supplementary file 1 [file Data_Sheet_1.docx]

Appendix A

Stimuli used in fMRI experiment

| Words | | Nonwords | |
| --- | --- | --- | --- |
| BLUE | KNEE | | TEG |
| BOOT | LAUGH | | SUT |
| CAPE | LYNX | | STET |
| CLAM | PEACH | | SITH |
| CREW | PEAR | | ROIN |
| EYE | PIE | | NINT |
| FLY | SEW | | MULP |
| FOOT | SHOE | | GEAD |
| FRUIT | SIGHT | | DOIL |
| GRAY | YACHT | | CHUT |

Supplemental Materials

| Clusters | Cluster Size | MNI Coordiates  [x, y, z] |
| --- | --- | --- |
| Left Angular Gyrus/Middle Occipital Cortex | 937 | [-42, -72, 22]  *[-42, -76, 30]*  *[-40, -60, 28]* |
| Primary Visual Cortex | 371 | [-14, -52, 16]  *[-12, -52, 8]*  *[-6, -62, 14]* |
| Precuneus | 188 | [0, -54, 10]  *[-4, -60, 20]*  *[-6, -50, -16]* |
| Right middle temporal gyrus | 144 | [50, -66, 10]  *[46, -68, 24]*  *[50, -58, 18]* |
| Left parahippocampal/fusiform gyrus | 132 | [-32, -42, -8]  *[-28, -48, -4]*  *[-30, -54, -10]* |
| Right calcarine | 93 | [8 -54, 10]  *[10, -60, 20]*  *[22, -58, 16]* |
| Right lingual gyrus | 91 | [20, -74, -6]  *[18, -82, -8]*  *[10, -86, -6]* |
| Right superior temporal/supramarginal gyrus | 88 | [60, -32, 24]  *[54, -36, 28]* |
| Right superior occipital gyrus | 67 | [18, -86, 20] |
| Right precuneus | 50 | [8, -58, 58] |

Table S1: Significant clusters of word > pseudoword contrast collapsing across all subjects (primary p < .001, Gaussian Random Field cluster-size threshold p_FWE_ < .05), including the MNI coordinate of the peak voxel and at most two other local maxima (> 8mm distance).

| Clusters | Cluster Size | MNI Coordiates  [x, y, z] |
| --- | --- | --- |
| Left inferior frontal gyrus/Insula | 2706 | [-34, 16, 12]  *[-56, 12, 30]*  *[-38, 4, 26]* |
| Right inferior frontal gyrus/Insula | 1253 | [-34, 16, 12]  *[-56, 12, 30]*  *[-38, 4, 26]* |
| Superior frontal gyrus (medial) | 856 | [-4, 20, 42]  *[-8, 8, 60]*  *[-2, 4, 66]* |
| Left inferior parietal lobule | 364 | [-50, -40, 58]  *[-56, -32, 50]*  *[-64, -18, 32]* |
| Midcingulate area | 250 | [0, -14, 28]  *[4, -34, 26]*  *[0, -24, 32]* |
| Right Cerebellum (lobule VI and crus I) | 179 | [26, -68, -24]  *[42, -68, -24]*  *[20, -72, -18]* |
| Right angular gyrus | 168 | [36, -68, 50]  *[36, -62, 56]*  *[26, -62, 46]* |
| Left inferior parietal lobule | 113 | [-28, -46, 42]  *[-34, -50, 48]*  *[-34, -52, 40]* |
| Right Thalamus | 79 | [8, -6, 6]  *[10, -10, 14]*  *[10, -8, -2]* |
| Right middle orbital frontal gyrus | 67 | [30, 62, -2]  *[36, 48, -10]*  *[40, 60, 0]* |
| Right superior orbital frontal gyrus | 64 | [24, 40, -12]  *[24, 50, -10]* |
| Left caudate and putamen | 52 | [-14, 8, 6]  *[-10, 0, 8]*  *[-20, 2, -4]* |

Table S2: Significant clusters of pseudoword > word contrast collapsing across all subjects (primary p < .001, Gaussian Random Field cluster-size threshold p_FWE_ < .05), including the MNI coordinate of the peak voxel and at most two other local maxima (> 8mm distance).

Figure S1. Sagittal slices of the regions that a significantly greater activation for pseudowords than words (red) or a significantly greater activation for words than pseudowords (blue) , using a primary p < .001, cluster-size threshold p_FWE_ < .05.


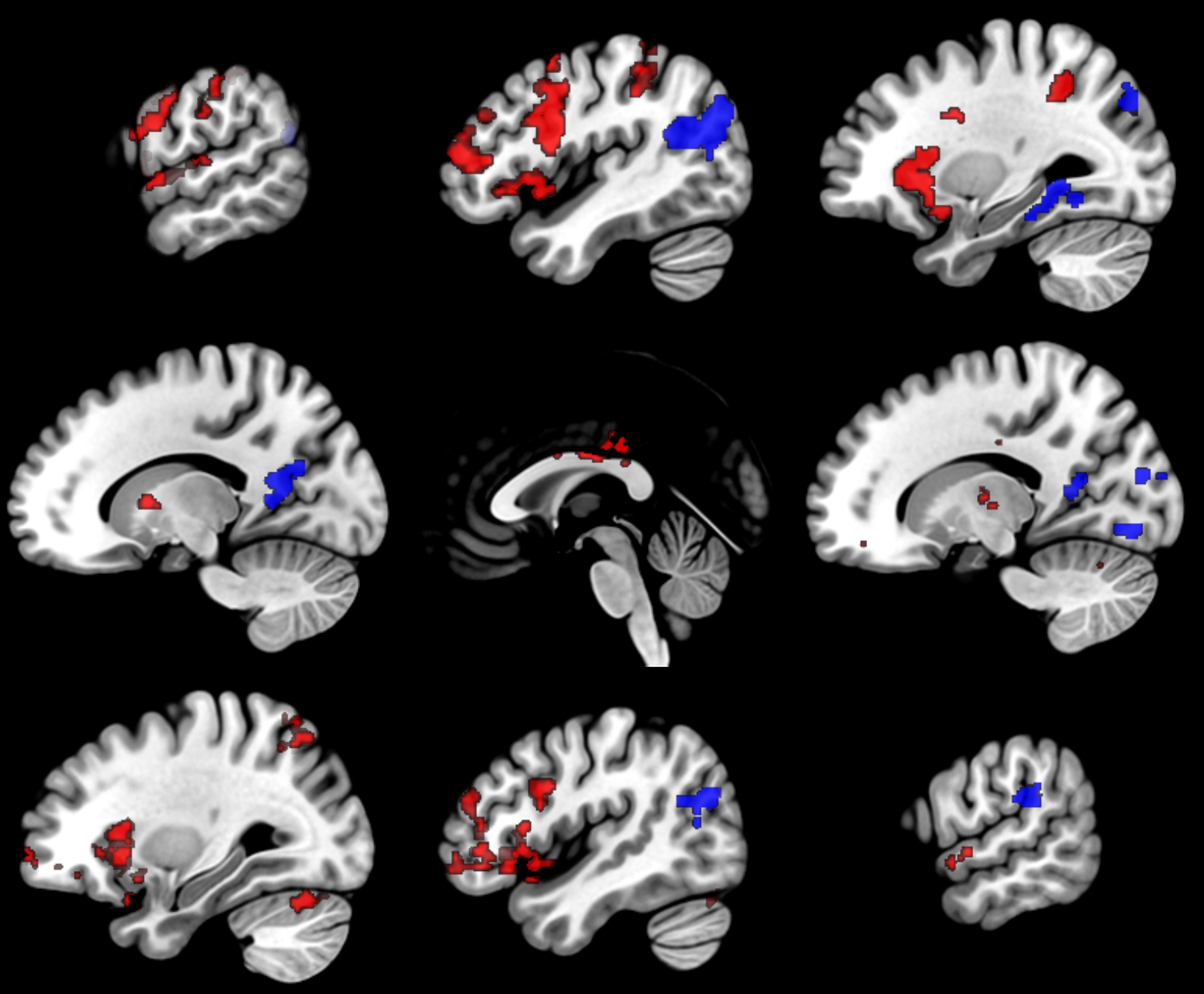


-60

-45

-30

-15

0

15

30

45

60
